# Postpartum dietary and physical activity-related beliefs and behaviors among women with recent gestational diabetes mellitus: a qualitative study from Singapore

**DOI:** 10.1186/s12884-021-04089-6

**Published:** 2021-09-07

**Authors:** Kailin Teh, Imm Pin Quek, Wern Ee Tang

**Affiliations:** 1 Kallang Polyclinic, National Healthcare Group Polyclinics, 701 Serangoon Road, Singapore, 328263 Singapore; 2 Yishun Polyclinic, National Healthcare Group Polyclinics, 2 Yishun Avenue 9, Singapore, 768898 Singapore; 3 Clinical Research Unit, National Healthcare Group Polyclinics, 3 Fusionopolis Link, Nexus @ one-north, Singapore, 138543 Singapore

**Keywords:** Gestational diabetes mellitus, Postpartum, Lifestyle, Maternal health, Qualitative

## Abstract

**Background:**

A woman with a history of GDM has at least seven-fold increased lifetime risk of developing type 2 diabetes mellitus (T2DM), compared to women who have normoglycemic pregnancies. Postpartum lifestyle modification has been shown to reduce postpartum weight retention and prevent the progression to T2DM. The aim of this study was to explore the postpartum dietary and physical activity-related beliefs and behaviors among women in Singapore who had GDM in their most recent pregnancies.

**Methods:**

Semi-structured in-depth interviews were conducted with 14 women, who were up to four months postpartum and had GDM in their most recent pregnancies. Interview data were analyzed using thematic analysis.

**Results:**

Three themes were identified in the analysis: (1) risk perception and knowledge regarding future diabetes, (2) suboptimal diet and physical activity after delivery and (3) factors influencing the postpartum lifestyle.

**Conclusions:**

The study findings provided useful information on the postpartum lifestyle beliefs and behaviors among women with a history of GDM. Most participants had low risk perception of future diabetes and their diet and physical activity after delivery were suboptimal due to various influences. These insights can be used to design tailored materials and programs to support women who have had GDM reduce their risk of developing future T2DM.

## Introduction

Gestational diabetes mellitus (GDM) is associated with a higher risk of maternal type 2 diabetes mellitus (T2DM), metabolic syndrome and cardiovascular disease later in life [1, 2]. In particular, a history of GDM increases a woman’s lifetime risk of developing T2DM by at least seven-fold, compared to women who have normoglycemic pregnancies [3]. The rates of GDM and T2DM are increasing globally, placing a great economic burden on the healthcare system [4]. Singapore has one of the highest prevalence of GDM worldwide, with approximately 21% of pregnancies complicated by GDM using the 2013 World Health Organization (WHO) criteria for GDM diagnosis [5]. An estimated 25% of women with GDM could develop pre-diabetes or T2DM within 5 years [6].

Pregnancy is often described as a 'teachable moment' to promote health behavior change as the risk perception of most women may increase during this special phase of life, and hence they may be more motivated to reevaluate their health needs and redefine their priorities [7]. The diagnosis of GDM spurs many women to engage in a healthy lifestyle during pregnancy due to concerns of obstetric risks and complications. However, the behavior change is often not sustained as they lose their motivation after giving birth [8]. Studies have shown that many women with a history of GDM have increased caloric intake, suboptimal levels of physical activity, increased post-pregnancy weight and body mass index (BMI) after delivery [9–12], which also increases the risks of postpartum T2DM and prediabetes [13].

The postpartum period is a time of vulnerability for most mothers, as they experience many physiological, emotional, and social changes. In addition to coping with competing demands of motherhood, work and family commitments [14], many Asian women practise confinement and need to observe a set of traditional customs and practices to assist them to recover from pregnancy and childbirth. For example, a special diet is taken to improve personal health and promote milk supply. There are restrictions on personal hygiene such as no bathing or bathing with herbal preparations. Women also rest at home and avoid strenuous physical activities to regain their strength and prevent future illness. Some will engage a confinement nanny to help with household chores and care of the baby [15–17].

Women with a history of GDM represent a high-risk population identified at an early stage when they are eligible for health interventions. Beyond pregnancy, the postpartum period is an important window of opportunity for healthcare professionals to provide advice and support to these women as lifestyle modification, such as healthy diet and increased physical activity, has been shown to prevent further progression to T2DM [18–20]. There is a need to understand their postpartum lifestyle, which may be influenced by confinement practices, and identify any unhealthy behaviours.

To date, studies related to GDM conducted in Singapore were mostly focused on the prevalence, diagnosis criteria, antenatal and postnatal screening. No study on the postpartum lifestyle beliefs and behaviors among women with GDM, especially during the confinement period, in Singapore has been found. Hence this study was embarked with the aim to explore the dietary and physical activity-related beliefs and behaviors during the first four months post-delivery among women in Singapore who had GDM in their most recent pregnancies. These insights can potentially aid in tailoring culturally-sensitive postpartum care and guiding interventions to prevent diabetes for women with a history of GDM in Singapore’s multi-ethnic society.

## Methods

### Design

We chose a qualitative descriptive research design with the use of semi-structured in-depth individual interviews to explore the postpartum dietary and physical activity-related beliefs and behaviors among women with a history of GDM in Singapore. Though this methodology has been regarded by some researchers as less theoretical compared to other qualitative designs, it proved to be a valuable approach in our study as it allowed the investigators to study the topic in its natural state, remain close to the data, gain an accurate and straight understanding of the participants’ experiences and perceptions on their diet and physical activity at different time points after delivery, and focus on describing the details, without being restricted by conceptual or philosophical assumptions [21–23].

### Participants

We sampled participants purposively to achieve a mix of characteristics among the participants in relation to age, ethnicity, educational level, socioeconomic background, parity and number of GDM pregnancies, with the final recruitment number determined by achievement of data saturation [24, 25]. Women aged 21 years and above, who are Singapore citizens and Permanent Residents, with a self-reported history of GDM in their most recent pregnancies and up to four months postpartum were included in this study. Those with pre-existing T2DM were excluded.

Potentially eligible participants were first identified by healthcare professionals working in two public primary care clinics in Singapore, then referred to the investigators. The investigators contacted 53 women by phone, screened them for eligibility, and arranged face-to-face interviews with those who agreed to participate. Thirty-eight women refused to participate as they were not interested (n=33) or were not Singapore citizens or Permanent Residents (n=5). Fifteen women provided consent to participate but one was subsequently withdrawn due to a diagnosis of pre-existing T2DM. Therefore data collected from 14 women were included in the data analysis.

### Data collection

Data were collected from August 2018 to May 2019 through individual face-to-face semi-structured in-depth interviews. A questionnaire was given to each participant before the start of the interview to collect her socio-demographic information, medical and obstetric history. Each interview was conducted by KT who is a Family Physician with special interest in women’s health, or IPQ who is a women’s health Advanced Practice Nurse. Both received training in conducting qualitative interviews and were not known to the participants before the interviews.

We designed an interview guide with reference to existing literature and discussion among the investigators. A sample of the main interview questions is provided in Table 1. The interview guide consisted of questions on experiences with GDM, confinement practices, beliefs, experiences, perceived barriers, and facilitators of a healthy diet and physical activity. We pilot-tested the interview guide with two healthcare professionals and one participant and modified the interview guide iteratively over the course of the study based on previous interviews. The participants were given the opportunity to discuss freely based on the questions asked. Probes and follow-up questions were used to facilitate discussion.

**Table 1:** Interview guide

Main interview questions	
1. What was the experience of having GDM like for you?	
2. What do you think about the impact of GDM on your health in the future?	
3. Please describe to me your current lifestyle on a typical day.	
4. After delivery, are there any specific tradition or practices that you do or do not do in your daily life?	
5. What are your views on a healthy lifestyle for women after delivery?	
6. Please tell me more about your current diet on average in a day.	
7. What are the challenges you face that make it difficult for you to improve your current diet?	
8. Is there anything that you feel can help you improve your diet?	
9. On average, how much time do you spend on physical activity in a week currently?	
10. What are the challenges you face that make it difficult for you to engage in more physical activities after delivery?	
11. Is there anything you think can help you engage in more physical activities after delivery?	
12. What concerns do you have about your weight?	

Each interview was conducted in English in a comfortable non-clinical meeting room and lasted 52 minutes on average. Repeat interviews were not carried out. The interviews were digitally audio-recorded and transcribed verbatim with written consent from the participants. Relevant notes and observations were recorded after each interview, including the behavior and demeanor of the participant, non-verbal communication during the interview, and the investigator’s thoughts and interpretations of the conversations, to complement the transcripts during data analysis.

### Data analysis

Data analysis was conducted by both investigators and performed simultaneously with data collection to enable the incorporation of emerging themes into subsequent interviews. The six-step thematic analysis framework was used as it is not linked to a specific epistemological or theoretical position, ensuring that the investigators pay close attention to the experiences and perspectives of the participants throughout the analysis process [26]. The framework for ensuring trustworthiness in qualitative research proposed by Lincoln and Guba [27] was applied in this study.

To ensure credibility, investigator triangulation was used by involving both investigators in coding and analysis. The investigators first read through the transcripts closely to familiarize themselves with the data. Next, independent inductive coding of the transcripts were conducted and a list of initial codes was generated. The third, fourth and fifth steps involved identifying and reviewing themes among associated codes, followed by refining, defining and naming the themes. Any differences regarding codes and themes were discussed by both investigators until consensus was reached. During coding, both investigators documented their reflections to evaluate how their own views influenced their interpretation of the data. Lastly, the themes generated were compiled with representative quotes to illustrate the findings.

Transcripts and findings were not returned to the participants as they represented an interpretation of the synthesis of all the interviews and not individual participants [28]. The consolidated criteria for reporting qualitative research (COREQ) guidelines were used in this study.

## Results

### Participant characteristics

A summary of the demographic characteristics of the 14 participants is presented in Table 2. The mean age was 34.3 years (range from 28 to 43 years). Most were Chinese and had completed undergraduate degree. The participants had a mean number of 1.6 children (range from one to three children) and were interviewed at an average of 80.9 days (range from 43 to 134 days) post-delivery. Most had one previous GDM pregnancy (*n*=10). The mean postpartum BMI was 22.6 kg/m^2^ (range from 16.2 to 30.5 kg/m^2^).

**Table 2:** Participant characteristics, *N* = 14

Characteristic	n (%)
**Marital status**	
Married	14 (100)
**Ethnicity**	
Chinese	9 (64.3)
Malay	2 (14.3)
Indian	2 (14.3)
Others (Burmese)	1 (7.1)
**Education**	
Completed secondary school	2 (14.3)
Completed diploma degree	1 (7.1)
Completed undergraduate degree	11 (78.6)
**Employment status prior to delivery**	
Homemaker	2 (14.3)
Working part time	1 (7.1)
Working full time	10 (71.4)
Self-employed	1 (7.1)
**Housing type**	
HDB 3-4 room flat	6 (42.9)
HDB 5 room flat/Executive flat	3 (21.4)
Condominium / Landed property	5 (35.7)
**Place of delivery for the most recent pregnancy**	
Public hospital	7 (50.0)
Private hospital	7 (50.0)
**Parity**	
1 child	7 (50.0)
2 children	5 (35.7)
3 children	2 (14.3)
**Number of GDM pregnancies**	
1	10 (71.4)
2	3 (21.4)
3	1 (7.1)
**First-degree family history of DM**	
Yes	5 (35.7)
No	9 (64.3)
**Postpartum BMI, kg/m**^**2**^	
< 18.5	2 (14.3)
18.5 to 22.9	6 (42.9)
≥ 23.0	6 (42.9)

### Identified themes

Three key themes were identified through interviews of these 14 participants, including:

1. Risk perception and knowledge regarding future diabetes
2. Suboptimal diet and physical activity after delivery
3. Factors influencing the postpartum lifestyle

#### Theme 1: Risk perception and knowledge regarding future diabetes

Most participants, including those with more than one previous GDM pregnancies and those with higher education level, did not perceive themselves as having higher risk of developing T2DM in the future. They believed that GDM is a transient health condition and that health ensues after getting normal results for the postnatal oral glucose tolerance test (OGTT).

*GDM is just like, oh it was a temporary condition. So, after you give birth, it goes away. And then, kind of revert to premorbid lifestyle. (Participant 7, 27 years old, para 1)*

*Because I’ve cleared my OGTT, so I didn’t feel the need to do anything already. (Participant 2, 42 years old, para 3)*

In addition, a positive family history of diabetes mellitus was perceived to be a more significant risk factor of developing T2DM than a personal history of GDM. Some participants also believed that T2DM only occurs in older adults and they were currently at lower risk of developing the condition.

*I think because for me, I have a family history of diabetes, so I know that you need to keep on monitoring. (Participant 9, 33 years old, para 2)*

*Maybe because I’m still young, so my mentality is like, I might not get it. But as I grow older, maybe. (Participant 8, 28 years old, para 2)*

The participants reported getting some advice on healthy lifestyle during their GDM pregnancies. However the advice was mainly focused on the importance of staying healthy to prevent obstetric complications of GDM, and the risk of future diabetes was only briefly mentioned. Furthermore, they did not receive clear instructions on transfer of care from the tertiary institution to primary care to continue follow-up after delivery. The participants did not receive specific information or counselling related to postpartum self-management of health from healthcare professionals. This in turn perpetuated their low self-perceived risk of developing diabetes. Most participants shared the same sentiment as Participants 7 and 8, and no longer felt the need to maintain the healthy lifestyle that they had adopted during pregnancy in the postpartum period.

*Post-delivery I was not given any information, I wasn’t sure whether I still need to keep to that strict diet regime that was given to me, but I didn’t bother. Because I already gave birth, so I just started eating what I wanted. (Participant 7, 27 years old, para 1)*

*Already done my part, I gave birth to the baby, so in terms of what I ate would not greatly affect my baby… I think there’s a bit more liberty already after you give birth… (Participant 8, 28 years old, para 2)*

With lack of health advice from healthcare professionals, participants turned to platforms such as Google, internet forums and social media sites to look for information on how to cope during and after a GDM pregnancy. Some expressed their uncertainty of the credibility of the information whereas other searched in vain for resources specific to postpartum care for GDM mothers.

*The moment I found out that I was GDM, I was like googling like crazy to find out how I can control my GDM and what causes GDM, you know that kind of thing. (Participant 15, 30 years old, para 1)*

*I just blast on my Instagram... They are just the random followers...After the lady talked to me about GI and all that, then I had a direction on what I’m supposed look for. So I started to eat low GI stuff. (Participant 13, 35 years old, para 1)*

Participant 6 shared her disappointment with the lack of postpartum support from healthcare professionals and described how she relied on constant self-talk as a reminder to stay healthy so that her chance of developing T2DM would be reduced.

*I think as a nature of human; you tend to forget things after a while. You tend to forget, oh you have GDM, GDM actually increases the risk of having diabetes. Most of the time, you will think, ok now it’s over already. I am back to normal. My OGTT result is normal now. I can slack a bit. I think we need a constant reminder that you need to be really compliant to the healthy lifestyle. (Participant 6, 29 years old, para 1)*

#### Theme 2: Suboptimal diet and physical activity after delivery

Overall, unhealthy dietary behaviors in the postpartum period were commonly reported by the participants although they came from varying backgrounds and differed in the number of previous GDM pregnancies. They had lack of knowledge on the concept of My Healthy Plate, which was introduced by the Singapore Health Promotion Board (HPB) to guide Singaporeans on planning a healthy meal. Only one participant was following My Healthy Plate correctly in her postpartum meals.

All participants practiced confinement ranging from 14 to 40 days after delivery, during which they were consuming food prepared by a confinement lady or caterer. The confinement diet was similar in all participants across different ethnicities. It mainly consisted of fish, seafood, chicken and vegetables cooked with more “heating” food (e.g. ginger, wine and sesame oil) which were believed to aid in postnatal recovery. Participants avoided “cooling” food and consumed herbal soups and red date tea to keep the body “warm” and restore the balance upset by childbirth.

*Must eat food, confinement food, very important to bring back the nutrition into your body. (Participant 9, 33 years old, para 2)*

*The first two weeks, there’s a lot of herbal soups… for the womb to heal, so that it will expel out the excess blood. (Participant 15, 30 years old, para 1)*

Most participants reported excessive consumption of refined carbohydrates, for instance white rice, white bread, pasta, crackers, pastries and sweetened beverages, especially after their confinement period during which they opted to dine out or prepare their own meals. Their postpartum diet also lacked the recommended intake of fruit and vegetables. This unhealthy dietary habit was more prominent among participants who often experienced increased hunger during breastfeeding.

*I was so hungry initially when I was breastfeeding, it killed me... I did eat more carbs after I delivered. I think it was the hunger… (Participant 3, 33 years old, para 2)*

The participants also admitted that indulgence in cravings post-delivery after being deprived of their favourite foods during a GDM pregnancy gave them pleasure and satisfaction, which helped them cope better with stress of motherhood.

*It went back right after delivery. My gynae came in and then, she saw two bags of chips by my bed. Then she said “Oh, you’re already eating this?” I told him after delivery, I want my bak chor mee (minced meat noodles), I want everything, everything that I have been avoiding… I don’t believe in limiting diet because I feel like it makes you miserable. So, I think you should eat whatever that makes you happy, as long as it’s not cancer-causing. (Participant 12, 31 years old, para 1)*

The Singapore HPB recommends that adults should do at least 150 minutes of moderate intensity physical activity a week to achieve substantial health benefits. Unawareness of the guidelines was a prominent finding during the interviews. The participants had suboptimal level of physical activity in the postpartum period, and most of them were not as active as they had been during pregnancy.

*I didn’t do much physical activities. Just the regular walking, you know, taking care of the baby… I do go for walks (before pregnancy). But so far in these two months, because I have to manage the baby, I haven’t had a chance to… (Participant 4, 34 years old, para 1)*

Walking was a preferred activity as it was considered by the participants to be the most convenient and suitable for their hectic lifestyle. They engaged in walking at a low- to moderate-intensity pace and most incorporated it into their daily routines, such as taking the stairs instead of the lift, walking to the grocery store and dog walking. Housework such as doing laundry and mopping the floor was another frequently performed physical activity among the participants.

*To be honest, I don’t have time to really exercise, it’s just really walking… I wish I have time to go for yoga, swimming and all those, but the lifestyle now is quite tight. (Participant 11, 38 years old, para 3)*

*You do a lot of housework and you really sweat, especially mopping, taking things, all these are exercise. (Participant 13, 35 years old, para 1)*

#### Theme 3: Factors influencing the postpartum lifestyle

##### Role as a mother

Motherhood exhaustion was commonly reported by both first-time and experienced mothers during the interviews, with top barriers to adopting a healthy postpartum lifestyle being lack of time and fatigue. They often had irregular meals due to the need to feed and attend to the baby and preferred taking a diet convenient for their schedule. When they had some spare time, getting rest and sleep was considered to be more important than exercise.

*No choice, I couldn’t really take care much of myself because I had to take care of my newborn, my son and all. So, it was quite tough… I forget that I’m hungry, I need to munch. I just lost track of time; I just keep doing things... You are physically and mentally drained and you don’t have rest. Sometimes, you have to eat faster because the child will cry for milk, or you don’t know why they are crying. You don’t even have proper time to eat. (Participant 8, 28 years old, para 2)*

*I feel that resting is much more important than exercise… I really don’t have enough sleep, how to think of exercise. (Participant 1, 40 years old, para 2)*

The participants described that as mothers, they usually put their own health needs as the lowest priority after delivery. They shared their struggle with the need for perfection in motherhood and expressed that they simply had no time to think of self-care.

*There is a lot of pressure on mothers to be the perfect mother… After you deliver, you have to care for the baby, and if you don’t have help for the housework, you also have to do the housework and then cooking... You know, in the end, the last priority will be yourself. (Participant 9, 33 years old, para 2)*

*I know that we need to take care of ourselves, but we put ourselves second and we put the baby first. So it is difficult to get enough rest or proper food… You need to be healthy, but making those changes is a bit difficult at the moment, after delivery. (Participant 14, 30 years old, para 1)*

##### Concern about the baby’s health

Concerns about their children’s nutrition and health was another frequently reported reason for adopting a healthy diet after delivery among most participants. They would try to eat healthy foods and have a balanced diet to improve the breast milk quality.

*Now I’m more conscious in eating more nutritious food. Previously, before my pregnancy, I just eat whatever I like. Now I’m more conscious, to make sure the baby grows more healthily… whatever I take, you know, it becomes the milk to feed him. (Participant 5, 34 years old, para 1)*

In addition, there was fear of reduced breast milk production if they restricted their diet. They believed that carbohydrate intake was important to ensure sufficient breast milk supply, hence carbohydrate was quoted to be an essential component of their diet.

*They say you need to eat well in order to produce milk. If you don’t eat properly, the milk will get lesser. (Participant 8, 28 years old, para 2)*

The participants also shared their worries about their children having a higher risk of developing diabetes when they grow up in view of their history of GDM. Participant 1 started paying more attention to the food she consumed as she realized that her dietary habits may influence that of her children.

*I think for GDM mothers, the chances of their children having diabetes will be higher. So I have to control my children’s diet. In order to control their diet, I have to control my diet also. (Participant 1, 40 years old, para 2)*

The participants expressed their concerns about going outdoors to exercise with their baby. They were worried about the baby being bitten by insects and getting uncomfortable due to the hot weather in Singapore. They were not confident in handling the baby alone when going out. This was illustrated by quotes such as:

*It is actually really hot, it is just very warm, so I don’t bring him out, especially because he gets sweaty very easily and he gets bothered by it. (Participant 7, 27 years old, para 1)*

##### Traditional postpartum practices

Certain confinement beliefs and practices had a negative influence on the postpartum lifestyle of the participants. For example, the participants were consuming red date tea frequently in place of plain water after delivery as red date was thought to help boost the immune system and replenish blood. They were unaware of its high sugar content and did not limit their intake. A Chinese participant shared that she was advised by her parents-in-law to avoid taking fruits during confinement which were perceived to be “cooling” in Chinese cultural beliefs. The Indian participants expressed their difficulties in eating healthily as their postpartum meals followed their traditional diet which tended to be unhealthier in terms of seasoning and cooking method. Some participants also reflected that they were supposed to minimize physical activities and stay at home during confinement, in order to get rest and recover from the delivery.

*My mother in law doesn’t allow me… I was doing some yoga stretches, then she was like, what are you doing?! She said I cannot engage in all these activities for the first month, the womb will come down, this kind of thing… (Participant 9, 33 years old, para 2)*

##### Family support

Family influences on adopting a healthy postpartum lifestyle were both positively and negatively reported by the participants.

Health-conscious and supportive spouses who reminded participants constantly to eat healthily and accompanied them on walks for exercise were viewed as a strong motivational factor to maintain a healthy lifestyle after delivery. The participants’ mothers were also an important source of encouragement and provided helpful advice on appropriate foods and cooking methods. Participant 6 highlighted that she was appreciative that her whole family was willing to change to a healthier diet for her sake during pregnancy and continued after delivery.

*You definitely need some support from your family. You need somebody that really can sync the same thought with you, so that you can continue with your healthy lifestyle. (Participant 6, 29 years old, para 1)*

Some participants found it time-consuming and difficult to prepare separate meals for themselves, their spouses and families. Hence, they tended to choose food options based on their spouses’ and families’ food preferences. Participant 8 shared that her spouse was not health-conscious and led an inactive lifestyle. This resulted in a negative influence on her dietary and physical activity behaviors.

*My husband is not the person who likes to have this kind of healthy lifestyle. So it’s quite difficult for me because he’s a very fussy eater, he doesn’t eat certain foods… if I cook for myself, and then cook for him, it’s quite tough... (Participant 8, 28 years old, para 2)*

Babysitting by the spouse and other family members was also felt to be a crucial factor which allowed participants personal time to engage in more physical activity in the postpartum period.

##### Food environment

Easy access to junk food and sweetened beverages, higher cost of healthy eating and limited food options when ordering from food delivery platforms or dining out made it challenging for the participants to adhere to a healthy diet. They also had difficulty controlling their food portions and choosing a balanced meal according to My Healthy Plate when dining out.

*I think it is the wealth of things we have. We have an abundance of choices. Everywhere you go, the temptation, the abundance of cakes, fruits, chocolates. So that is the challenge. (Participant 4, 34 years old, para 1)*

*It can be quite expensive though, eating healthy in Singapore. You want people to cut down on all that diabetes, but your healthy options are not cheap. If you want to eat a salad bowl, it is probably 6, 7 dollars. And then you can get a McDonald meal for cheaper. (Participant 14, 30 years old, para 1)*

##### Intrinsic motivation

The participants shared their struggles with their postpartum weight retention and felt the pressure to reduce their weight to pre-pregnancy weight, if not better. The desire to improve the body image after delivery was the main motivation to modify their lifestyle for most participants.

*I think exercise is good for you anyway. And for me, I had a very big belly by the time I delivered… So, me wanting to go back to exercise is for me, not because of GDM per se… (Participant 3, 33 years old, para 2)*

*To look good, to feel confident, so that I can have that mood to go out. I feel like all the clothes don’t fit me, I don’t know, I feel a bit upset. (Participant 7, 27 years old, para 1)*

Some participants also voiced their desire to lead a healthy lifestyle and maintain fitness in the long run, so as to reduce the overall risk of chronic diseases, including T2DM, which are highly prevalent in Singapore due to an ageing population.

*Overall, not just weight management, I think diet management and exercise all come together. If not, the risk is getting into a type 2 diabetes. (Participant 2, 42 years old, para 3)*

When asked about the role of diet versus physical activity in staying healthy, they believed that dietary control plays a more important role than exercise and is easier to be achieved in the first few months postpartum as most mothers would not have time to exercise.

*After you delivered your baby, I guess most mummies don’t have much time for physical activity for the first few months. So, I think diet does play a very important role. (Participant 6, 29 years old, para 1)*

Despite having concerns about their personal health, most participants reported lack of self-discipline in adhering to healthy lifestyle practices. They experienced difficulty resisting junk food cravings and overcoming inertia to participate in physical activities.

*I see them (husband and children) lazing at home, then I’m like, why should I go run, ok I’ll wait for you when you all are ready to go… To even get changed to get out to run is a discipline on its own! I think it’s personal, the hurdle is personal… (Participant 2, 42 years old, para 3)*

## Discussion

In this study, discussion with participants with a history of GDM through interviews provided an insight into their knowledge, experiences and practices regarding diet and physical activity during the first four months postpartum. The study indicated that women with GDM had low risk perception of developing T2DM in the long run. Despite having varied backgrounds, the participants shared similar behaviors of unhealthy eating and insufficient physical activity after delivery. Barriers and facilitators of a healthy postpartum lifestyle were not directly related to the study aim; however, they were frequently mentioned by the participants when they were describing and justifying their health behaviors.

### Low risk perception and lack of knowledge regarding T2DM

Most participants perceived themselves to be at low risk of T2DM after a GDM pregnancy, which was similar to the findings of other studies [29–31]. This low risk perception of future development of T2DM could be one of the underlying reasons for having unhealthy postpartum lifestyle behaviors.

Risk perception plays a crucial role in motivating health behavior change to produce positive health outcomes. It may be influenced by many factors such as personal experiences, amount of information available to an individual, the frequency with which a threat is represented in the media and family history of the disease [32].

These influencing factors were evident in this study. Mothers with more than one GDM pregnancies assumed that they would ‘clear’ each postnatal glucose screening test and that their health would be back to normal after delivery. During pregnancy, these women had regular contact with healthcare professionals. However, the level of support for self-management varied among the participants and information given to the participants during antenatal care was focused on adopting lifestyle changes with the main aim of preventing obstetric complications of GDM. Moreover, in the postpartum period, there was limited advice regarding appropriate lifestyle practices from healthcare professionals and inadequate reinforcement of the message of GDM being a risk factor for developing T2DM, which was a finding also highlighted by other studies [33, 34]. This suboptimal follow-up care may downplay the significance of future health risks of GDM. The reassurance from healthcare professionals after a normal postnatal glucose screening test further perpetuated the perception of GDM being a transient condition during pregnancy without any long-term health implications. This study had clearly identified a missed opportunity to educate these women to make lifestyle changes and maintain them beyond the pregnancy.

Although the Singapore government had launched a “war on diabetes” since 2016, there is generally a lack of exposure on GDM and its associated risks in the media. Participants felt isolated by the diagnosis and had limited knowledge of the future health risks of GDM, even after attempting to read from online resources on their own initiative. This had resulted in limited awareness of the risks of GDM, reduced risk perception and inability to engage in self-management in the postpartum period among participants, including those with higher education level and socioeconomic status. This study showed that education on the risk of diabetes and the importance of healthy living must be reinforced during the postpartum and long-term follow up, and not only during the antenatal period for this high-risk population. It is necessary to increase awareness among healthcare professionals regarding the need to provide continuous education and information on credible resources for these women.

### Unhealthy eating and insufficient physical activity post-delivery

Despite having varied backgrounds, the participants shared similar behaviors of unhealthy eating and insufficient physical activity after delivery. It was apparent from the interviews that while the diagnosis of GDM motivated the participants to modify their diet and physical activity during their pregnancy in order to have an uneventful delivery and a healthy baby, these changes were often not sustained after delivery. This drop-off in positive health behavior was supported by two other reviews which reported suboptimal lifestyle behaviors during the postpartum period [35].

Consistent with other studies [10, 11, 36, 37], most participants in this study had unhealthy dietary behaviors after delivery. Interestingly, this observation was more prominent after their confinement period. During confinement, the participants had fairly controlled portions and balanced food types in their meals which were specially prepared by others. After the confinement period ended, many participants had to cook themselves or dine out, resulting in an increased consumption of refined carbohydrates and sugar intake and a marked reduction in consumption of vegetables and fruits. This may possibly speed up their trajectory towards development of T2DM. These findings suggested that refresher educational courses or workshops on dietary and lifestyle modification in the postpartum period might be useful to reinforce the existing knowledge of healthy lifestyle learnt during pregnancy and maintaining it in the long-run for women with a history of GDM.

There is evidence in the literature stating the challenges encountered by patients to make dietary changes due to reasons such as cultural beliefs and lack of healthier alternatives, especially those with lower socioeconomic status [38, 39]. In this study, participants shared some restrictions associated with traditional postpartum practices. It is therefore important to consider the cultural receptivity and feasibility among women of different ethnic groups who have GDM when making dietary recommendations and designing healthy recipes. Knowledge about cultural values and beliefs should be included in the training of healthcare professional to increase their cultural awareness and ability to provide appropriate care for these women. In addition, many Singaporeans prefer the convenience of eating readily available cooked food due to the hassle of preparing healthy meals at home. More national initiatives would be required to increase the accessibility and affordability of healthier meals and encourage Singaporeans to adhere to healthier food choices when they dine out or order from food delivery services.

Most participants considered themselves as active after delivery, but their reported level of physical activity in the postpartum period was actually suboptimal. Their activities were significantly reduced as compared to during pregnancy and this was echoed in other studies which indicated that women’s level of physical activity decreases after delivery [40, 41]. Walking and household chores were the most frequently reported physical activities among the participants. However the intensity at which these activities were performed varied widely. Women should be provided with clear information on the intensity of physical activity required to help them distinguish between the type of activities which can be counted towards the recommended guidelines of at least 150 minutes per week and activities which merely reduce sedentary time but do not meet the guidelines.

### Factors to consider for diabetes prevention interventions

Barriers and facilitators of a healthy postpartum lifestyle were frequently mentioned by the participants when they were describing and justifying their health behaviors. Since the postpartum period is unique with many challenges faced by mothers, identifying extrinsic and intrinsic influences is crucial to design behavior change interventions and develop diabetes prevention programs for women with a history of GDM.

The participants described the lack of time, fatigue and competing childcare responsibilities as their main barriers to engage in healthy lifestyle practices post-delivery, similar to previous studies [42–44]. In addition, the perceived obligation to achieve perfectionism in motherhood made it challenging for mothers to prioritize their own health and lifestyle changes over childcare or domestic duties. Asking for help was viewed by the participants as having poor parenting skills. These findings suggested that it would be important to include strategies that emphasize educating mothers on time management skills and self-care to assist these women to take on a position of responsibility for personal health and adopt healthy lifestyle behaviors.

The participants stressed the importance of social support in the postpartum period. Their spouses and families played a critical role in motivating them to eat healthily and engage in physical activities, which concurred with previous research, including a systematic review which confirmed that social support can increase one’s ability to change and retain new lifestyle habits [45, 46]. As such, it is essential for healthcare providers to understand the influence of spouse and family on one’s health and involve them early in lifestyle modification when managing women with a history of GDM. Proper counselling and health education should be provided to their spouses and families who can motivate and support the women in the postpartum period during which they often have reduced priority of self-care. To promote a sustained couple-based healthy lifestyle among these women, a holistic approach that extends beyond the individual to include the spouse for collaborative behavior needs to be employed in their postpartum care [47].

In addition, participants expressed dissatisfaction with receiving generic and vague health advice from their specialists during the antenatal and postpartum period. This finding highlighted the need to train healthcare professionals to provide tailored education and counseling on diet, physical activity and weight loss which suits one’s needs, lifestyle and culture. Pregnancy is often regarded as a 'teachable moment' to adopt positive health behaviors due to an increase in motivation towards health and frequent contact with the healthcare system. Healthcare professionals should take advantage of this motivation in women with GDM and introduce strategies that are grounded in theories of health behavior change such as the health belief model [48] and focused on increasing self-management to improve their health and maintain positive lifestyle behaviors beyond pregnancy. With adequate support and knowledge, these women may be able to better appreciate the importance of their future health and take a proactive role in healthy living to delay the progression of GDM to T2DM.

A systematic review of clinicians’ views on healthcare provision for women with recent GDM showed that opportunities to care for these women postnatally are missed due to insufficient communication [49]. This is also echoed in our study in which some participants missed their postnatal visits due to lack of follow-up instructions from the tertiary institutions. It highlighted a need for care coordination between tertiary institutions and primary care to ensure that women with a history of GDM have a seamless antenatal and postnatal follow-up and their health needs are addressed timely. A good time to reach out to mothers with GDM to reinforce the importance of the postnatal check and maintaining a healthy postpartum lifestyle would be before they are discharged from the hospital. When a woman presents to the primary care setting for her postnatal checkup, the family physician should also use the opportunity to educate her on the risk of future T2DM, provide tailored health advice and engage the family to adopt the same healthy lifestyle. Another potential area for health promotion and raising awareness is the child health services in primary care clinics. In Singapore, women usually visit these services regularly with their babies for immunizations and developmental assessments up to 4 years old. The nurses attending to these women could take the opportunity to motivate them to maintain a healthy lifestyle and continue long-term follow-up with their family physicians.

Mobile application may be a feasible method of disseminating health information and sending systematic reminders to women with a history of GDM to go for their postnatal check and regular glucose screening. Having ready and concise information in the phone would be more convenient for women to read at their own time, compared to printed materials and face-to-face workshops.

### Strengths and limitations

To our knowledge, this is the first qualitative study on the beliefs and behaviors of postpartum diet and physical activity among women with recent GDM pregnancies in Singapore. It is also unique as it described the influence of confinement on postpartum lifestyle in this population. The use of purposive sampling and in-depth interviews with information-rich cases provided valuable insights and better understanding of the perceptions and experiences after a GDM pregnancy among women from diverse backgrounds to inform future diabetes prevention interventions. The trustworthiness of the study was enhanced by striving to achieve credibility, transferability, dependability and confirmability.

This study has a number of limitations. The participants were all English-speaking and mostly Chinese. Hence, the findings of this study may not be transferable to the general population of women with a history of GDM in Singapore’s multi-racial society. The investigator who conducted the interview also performed the analysis which may introduce potential bias in interpretation. We attempted to mitigate the risk of bias through employing reflexivity and having another investigator who was not involved in the interview conduct data analysis independently. In this study, the interviews were conducted at up to four months postpartum and it was possible that the participants might have different lifestyle behaviors and beliefs at a later stage. Future research may be conducted with a longer follow-up period to observe their postpartum lifestyle.

## Conclusion

The findings of this study provided useful information on the postpartum lifestyle behaviors and health needs of women with a history of GDM in Singapore. These women had low risk awareness and perception of developing T2DM in the future. Their dietary and physical activity practices after delivery were far from optimal due to personal, family, cultural and environmental influences. These findings highlight the need for primary care and tertiary institutions to collaborate closely to increase the risk perception of future diabetes and improve the care for women with GDM, by providing them with timely support and culturally-appropriate educational information on postpartum dietary and lifestyle modification, with the ultimate aim to reduce their risk of ill health later in life.

## Data Availability

The datasets generated and/or analyzed during the current study are not publicly available due to individual privacy concerns but are available from the corresponding author on reasonable request.

## Abbreviations

GDM: Gestational diabetes mellitus
T2DM: Type 2 diabetes mellitus
